# Resilient distributed model predictive control for cooperative microgrids under communication loss with demand response integration

**DOI:** 10.1371/journal.pone.0345857

**Published:** 2026-04-08

**Authors:** Baheej Alghamdi

**Affiliations:** 1 Smart Grids Research Group, Center of Research Excellence in Renewable Energy and Power Systems, King Abdulaziz University, Jeddah, Saudi Arabia; 2 Department of Electrical and Computer Engineering, Faculty of Engineering, King Abdulaziz University, Jeddah, Saudi Arabia; Aalto University, FINLAND

## Abstract

This paper introduces a resilient distributed model predictive control (RDMPC) framework for coordinating energy management across networked microgrids with demand response integration. The coordination mechanism employs an alternating direction method of multipliers (ADMM)-based distributed MPC formulation that maintains tie-line reciprocity via a shared consensus schedule; standard ADMM convergence results apply under reliable communication for the convex quadratic-program relaxation. Safe operation under communication impairments and early termination is achieved by executing the reciprocal consensus tie-line setpoints and performing a local feasibility-repair step with physically interpretable slack variables (load shedding and spillage), providing anytime feasibility while (under reliable communication) optimality improves with additional ADMM iterations. Communication failure resilience is achieved by treating tie-line mismatch as bounded disturbances and applying two-sided reserve margins (upward and downward) through constraint tightening, ensuring feasibility for any mismatch within the assumed bounds when sufficient reserve headroom exists. Demand response is incorporated using distinct models for shiftable loads (energy-by-deadline) and curtailable loads (penalized reduction). Evaluation on a five-microgrid benchmark under packet loss, burst outages, and topology changes confirms feasible reciprocal execution under loss; relative performance versus naive distributed MPC (B2) is seed-dependent, and DR ablation shows large degradations (about 5.9× energy not served (ENS) and 5.8× cost increases) when flexibility is removed.

## Introduction

Microgrids have emerged as fundamental building blocks for modern power distribution systems, offering improved reliability, renewable energy integration, and local energy management capabilities [1]. The evolution toward interconnected multi-microgrid networks creates opportunities for resource sharing and economic optimization, but introduces coordination challenges [2].

Centralized Model Predictive Control (MPC) approaches, such as the seminal work by Ouammi et al. [3], achieve global optimality but suffer from critical vulnerabilities: single point of failure where communication loss with the central controller disables coordination entirely, scalability limitations as computational complexity grows with network size, and privacy concerns since all microgrids must share detailed operational data.

Distributed MPC (DMPC) addresses these limitations by decomposing the global problem into local subproblems [4]. However, existing DMPC methods typically assume reliable communication, whereas in practice communication networks experience packet loss, delays, and topology changes that can destabilize coordination.

The literature on MPC for microgrids spans centralized and distributed approaches. Ouammi et al. [3] developed a centralized MPC framework for five cooperative microgrids, reporting performance gains of about 4–9% (e.g., 4.4% and 8.5%) compared to single-microgrid operation, while Parisio et al. [5] extended MPC with explicit constraint handling. These works established MPC as the dominant control paradigm but retained centralized architectures. On the distributed side, Hans et al. [6] developed hierarchical DMPC for interconnected microgrids, and Bersani et al. [7] developed distributed robust control of power flows in cooperating microgrids. ADMM-based DMPC has gained particular attention for its convergence guarantees and decomposability [8]. Asynchrony and delays in ADMM-based coordination have also been studied, motivated by heterogeneous computation and communication times in distributed networks. Asynchronous ADMM variants provide convergence guarantees under partial asynchrony and delayed updates [9,10], establishing that stale information can be handled with appropriate parameter choices. In power-system optimization, ADMM-based distributed optimal power flow has been analyzed under stochastic communication delays [11] and asynchronous updates [12], supporting our modeling of packet loss during coordination iterations and our focus on feasible execution when coordination is incomplete.

Distributed demand response control strategies have been developed using Lyapunov optimization [13], though comprehensive integration of DR with resilient DMPC remains limited. Communication impairments have been studied in distributed optimization/control, including asynchronous ADMM schemes that tolerate delayed or stale updates [9,10]. Two consensus-based approaches explicitly handle packet losses in microgrid coordination [14,15], but neither specifies execution semantics under communication failure nor enforces tie-line reciprocity; explicit resilience mechanisms with feasibility guarantees and well-defined execution semantics for cooperative microgrids remain limited.

Several recent works address related aspects of distributed microgrid coordination. Ananduta et al. [16] developed ADMM-based DMPC for adversarial microgrid scenarios with detection-based resilience, though without explicit stochastic packet-loss modeling, explicit tie-line execution semantics under asymmetric loss, or two-sided reserve tightening for bounded mismatch. Pham and Ahn [17] compared ADMM and dual decomposition methods but did not address communication impairments. Xie et al. [18] applied tube MPC for forecast uncertainty but targeted renewable variability rather than tie-line mismatch from coordination failures.

Jia et al. [19] developed ADMM-based economic dispatch for frequency deviation but without communication loss modeling.

To the best of our knowledge, none of these works combine all three elements required for resilient execution: (i) communication loss modeling at both optimization and execution stages, (ii) explicit tie-line reciprocity enforcement via handshake contracts, and (iii) two-sided reserves for bounded mismatch. Our RDMPC framework uniquely integrates these elements with demand response as an additional resilience resource.

Despite significant progress, critical gaps remain in the literature. Most DMPC methods assume reliable communication without explicit resilience mechanisms. Coordination algorithms often lack convergence guarantees or anytime feasibility. Integration of demand response with distributed, resilient control is limited. Most importantly, feasibility under communication failures is rarely proven formally.

This paper makes three concrete technical contributions. First (C1), we propose an ADMM-based anytime DMPC coordination scheme with convergence guarantees under reliable communication for the convex relaxation; under packet loss we use a lossy-update variant and an execution policy that implements the consensus tie-line setpoint with local feasibility repair, ensuring network-feasible schedules at any iteration. Second (C2), we develop a resilience mechanism with bounded mismatch wherein communication failures induce neighbor exchange uncertainty modeled explicitly as bounded disturbances, with constraint satisfaction guaranteed via two-sided constraint tightening (upward and downward reserves) using tube MPC principles when sufficient reserve headroom exists. Third (C3), we integrate demand response with distinct flexibility types by including shiftable and curtailable loads in the distributed optimization, quantifying their value under failures, and demonstrating that DR compensates for lost coordination capability.

Across all scenarios, the intended design goal is safe and feasible execution under communication loss (including maintaining service by limiting ENS via the load-shedding slack penalty), even when consistent cost improvements relative to standard DMPC are not observed.

## Materials and methods

### Problem statement

Decision variables (for each microgrid *i* over horizon k=0,…,H−1) are:

- $P_{ch,i}(k),P_{dis,i}(k)\geq 0$: energy storage system (ESS) charging/discharging power [kW]
- $z_{i}(k)\in \{0,1\}$: Binary charge/discharge mode selector
- $P_{grid,i}^{+}(k),P_{grid,i}^{-}(k)\geq 0$: Grid import/export power [kW]
- $P_{shift,i}(k)\geq 0$: Shiftable load served [kW]
- $P_{curt,i}(k)\geq 0$: Curtailable load reduction [kW]
- $P_{spill,i}(k)\geq 0$: Renewable spillage/curtailment [kW]
- *P*_*ij*_(*k*): Power flow to neighbor *j* [kW] (positive = export from *i*)

Coupling across microgrids is enforced by tie-line reciprocity: $P_{ij}(k)+P_{ji}(k)=0$. This constraint couples neighboring microgrids’ decisions.

Information structure distinguishes local and shared information:

- *Local information*: Own ESS state, local loads, local renewable forecasts, local prices
- *Shared information*: Tie-line flow decisions with neighbors (via ADMM)

The objective is to minimize total network operating cost while satisfying all constraints, with coordination achieved through distributed optimization.

### Network topology

We consider a network of *N* microgrids represented as an undirected graph 𝒢=(𝒱,ℰ):

- 𝒱={1,2,…,N}: Set of microgrids
- ℰ⊆𝒱×𝒱: Set of power exchange links
- ${𝒩}_{i}=\{j:(i,j)\in ℰ\}$: Neighbors of microgrid *i*

**Fig 1:**
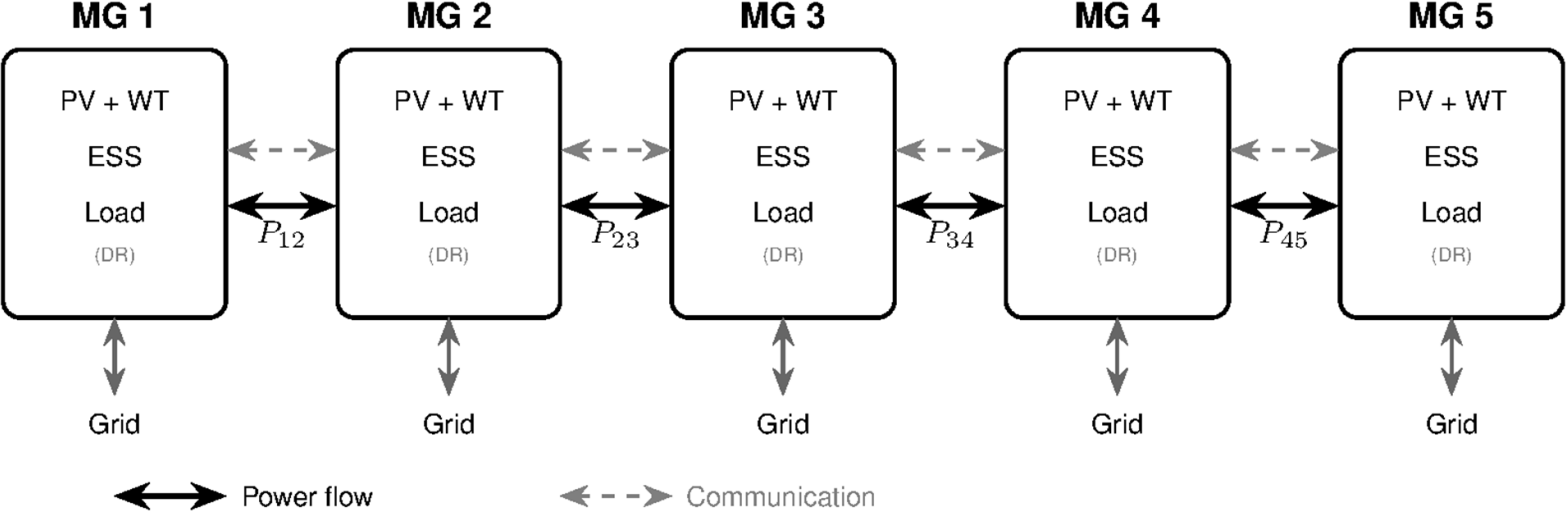
Five-microgrid network topology. Each microgrid has PV/WT generation, ESS, and DR-enabled loads. Solid lines show tie-line flows *P*_*ij*_; dashed lines show communication links.

The benchmark five-microgrid topology is shown in Fig 1.

### Microgrid components

#### Renewable generation.

Renewable power *P*_*res*,*i*_(*k*) is treated as a forecasted input to the MPC:


$${\hat{P}}_{res,i}(k)={\hat{P}}_{pv,i}(k)+{\hat{P}}_{wt,i}(k)$$ (1)

Forecasts are generated using probabilistic models (Weibull for wind [20], Beta for solar [21]) with scenario reduction [22,23] to obtain expected values. Renewable spillage (curtailment beyond planned):


$$0\leq P_{spill,i}(k)\leq {\hat{P}}_{res,i}(k)$$ (2)

The usable renewable power is:


$$P_{res,i}^{used}(k)={\hat{P}}_{res,i}(k)-P_{spill,i}(k)$$ (3)

### Energy storage system

State dynamics:


$$E_{i}(k+1)=E_{i}(k)+\eta_{c}P_{ch,i}(k)\Delta t-\frac{P_{dis,i}(k)\Delta t}{\eta_{d}}$$ (4)

where Δt [h] is the control interval.

Capacity constraints:


$$E_{i,min}\leq E_{i}(k)\leq E_{i,max}$$ (5)

Power constraints with binary mode selection (mixed-integer quadratic program (MIQP) formulation):


$$0\leq P_{ch,i}(k)\leq z_{i}(k)\cdot P_{ch,i,max}$$ (6)

$$0\leq P_{dis,i}(k)\leq (1-z_{i}(k))\cdot P_{dis,i,max}$$ (7)

$$z_{i}(k)\in \{0,1\}$$ (8)

This formulation correctly prevents simultaneous charging and discharging without nonconvex complementarity constraints. In the reported experiments, we relax $z_{i}(k)\in [0,1]$, yielding a convex QP. Relaxing *z*_*i*_(*k*) permits simultaneous charge/discharge in principle; in our experiments it was negligible (see Limitations).

Net ESS power:


$$P_{ess,i}(k)=P_{dis,i}(k)-P_{ch,i}(k)$$ (9)

### Demand response model

Total load consists of fixed, shiftable, and curtailable components:


$$P_{load,i}(k)=P_{fixed,i}(k)+P_{shift,i}(k)+P_{base,curt,i}(k)-P_{curt,i}(k)$$ (10)

Shiftable loads (e.g., electric vehicle (EV) charging, water heating) must receive their required energy by a deadline $k_{deadline}\leq H-1$ [24]:


$$0\leq P_{shift,i}(k)\leq P_{shift,i,max}(k)\;\sum_{\ell =0}^{k_{deadline}}P_{shift,i}(\ell )\Delta t=E_{shift,i,req}$$ (11)

We set *P*_*shift*,*i*,*max*_(*k*) = 0 for *k* > *k*_*deadline*_, so service does not occur after the deadline. The equality constraint ensures load shifting, not curtailment.

Curtailable load (can be reduced with penalty) satisfies:


$$0\leq P_{curt,i}(k)\leq P_{curt,i,max}(k)$$ (12)

We model the DR discomfort cost (linearized) as:


$$c_{dr,i}(k)=\lambda_{shift}\cdot d_{shift,i}^{+}(k)+\lambda_{shift}\cdot d_{shift,i}^{-}(k)+\lambda_{curt}\cdot P_{curt,i}(k)$$ (13)

where:


$$P_{shift,i}(k)-P_{shift,i,pref}(k)=d_{shift,i}^{+}(k)-d_{shift,i}^{-}(k)$$ (14)

$$d_{shift,i}^{+}(k),d_{shift,i}^{-}(k)\geq 0$$ (15)

### Grid exchange

Power exchange with the distribution network operator (DNO) using variable splitting:


$$P_{grid,i}(k)=P_{grid,i}^{+}(k)-P_{grid,i}^{-}(k)$$ (16)

$$0\leq P_{grid,i}^{+}(k)\leq P_{grid,i,max}^{+}$$ (17)

$$0\leq P_{grid,i}^{-}(k)\leq P_{grid,i,max}^{-}$$ (18)

In practice, simultaneous import/export is suboptimal due to spread ($\pi_{buy}>\pi_{sell}$), so the relaxation is tight.

### Tie-line exchange

Sign convention: *P*_*ij*_(*k*) > 0 means microgrid *i* exports to microgrid *j*.

Local bounds:


$$-P_{ij,max}\leq P_{ij}(k)\leq P_{ij,max}$$ (19)

Coupling constraint (enforced via ADMM):


$$P_{ij}(k)+P_{ji}(k)=0\;\forall (i,j)\in ℰ$$ (20)

### Power balance

For each microgrid *i* at each time step *k*:


$$P_{res,i}^{used}(k)+P_{dis,i}(k)-P_{ch,i}(k)+P_{grid,i}^{+}(k)-P_{grid,i}^{-}(k)-\sum_{j\in {𝒩}_{i}}P_{ij}(k)=P_{load,i}(k)$$ (21)

In implementation, we enforce the soft balance with slacks (Eq. (33)) to guarantee feasibility; Eq. (21) is recovered when $s_{i}^{LS}=s_{i}^{SP}=0$. Here *P*_*load*,*i*_(*k*) includes fixed, shiftable, and net curtailable components.

### Economic model

Time-varying electricity prices (assumed identical across microgrids for simplicity):

- $\pi_{buy}(k)$: Import price from DNO [$/kWh]
- $\pi_{sell}(k)$: Export price to DNO [$/kWh], where $\pi_{sell}(k)<\pi_{buy}(k)$

Instantaneous cost rate for microgrid *i* [$/h]:


$$\begin{array}{cc} c_{i}(k) & \;=\pi_{buy}(k)\cdot P_{grid,i}^{+}(k)-\pi_{sell}(k)\cdot P_{grid,i}^{-}(k)\\ & \;\;+\lambda_{ess}\cdot (P_{ch,i}(k)+P_{dis,i}(k))+c_{dr,i}(k)+\lambda_{spill}\cdot P_{spill,i}(k) \end{array}$$ (22)

where $\lambda_{spill}$ is a small penalty for renewable spillage (e.g., 0.01 $/kWh).

Operating cost over interval *k* [$]:


$$C_{i}(k)=c_{i}(k)\cdot \Delta t$$ (23)

### ADMM-based distributed MPC

#### Global optimization problem.

The centralized problem minimizes total network cost:


$$\min\sum_{i=1}^{N}\sum_{k=0}^{H-1}c_{i}(k)\cdot \Delta t$$ (24)

subject to all local constraints and coupling constraints from Eq (20).

#### ADMM formulation.

We reformulate using edge-based consensus variables. For each edge (i,j)∈ℰ, introduce $\phi_{ij}(k)$ as the consensus variable for tie-line flow. Although 𝒢 is undirected, we associate directed decision variables *P*_*ij*_ and *P*_*ji*_ with each undirected edge {i,j}∈ℰ.

Augmented Lagrangian:


$${ℒ}_{\rho }=\sum_{i=1}^{N}\sum_{k=0}^{H-1}c_{i}(k)\cdot \Delta t+\sum_{(i,j)\in ℰ}\sum_{k=0}^{H-1}\left[\mu_{ij}(k)(P_{ij}(k)-\phi_{ij}(k))+\frac{\rho }{2}{\left(P_{ij}(k)-\phi_{ij}(k)\right)}^{2}\right]$$ (25)

where $\mu_{ij}(k)$ is the dual variable [$/kW] and ρ>0 is the ADMM penalty parameter [p.u.].

#### ADMM iteration.

At each ADMM iteration *m*, each microgrid *i* solves the local subproblem:


$$\min_{u_{i}}\sum_{k=0}^{H-1}\left[c_{i}(k)\cdot \Delta t+\sum_{j\in {𝒩}_{i}}\left(\mu_{ij}^{\left(m\right)}(k)P_{ij}(k)+\frac{\rho }{2}{\left(P_{ij}(k)-\phi_{ij}^{\left(m\right)}(k)\right)}^{2}\right)\right]$$ (26)

subject to local constraints (ESS dynamics, DR constraints, power balance, bounds). Denote the resulting tie-line decision values at iteration *m* by $P_{ij}^{\left(m\right)}(k)$.

Next, for each edge (*i*,*j*), the consensus update is:


$$\phi_{ij}^{\left(m+1\right)}(k)=\frac{1}{2}\left(P_{ij}^{\left(m\right)}(k)-P_{ji}^{\left(m\right)}(k)\right)$$ (27)

Under packet loss, the consensus and dual updates are applied only when the bidirectional handshake succeeds ($h_{ij}^{\left(m\right)}=1$); otherwise $\phi_{ij}$ is held and $\mu_{ij}$ is frozen (Algorithm 1).

Note: $\phi_{ij}=-\phi_{ji}$ by construction, ensuring network-feasible tie-line setpoints.

Finally, the dual update is:


$$\mu_{ij}^{\left(m+1\right)}(k)=\mu_{ij}^{\left(m\right)}(k)+\rho \left(P_{ij}^{\left(m\right)}(k)-\phi_{ij}^{\left(m+1\right)}(k)\right)$$ (28)

### Convergence analysis

Primal residual:


$$r^{\left(m\right)}=\sqrt{\sum_{(i,j)\in ℰ}\sum_{k=0}^{H-1}{\left(P_{ij}^{\left(m\right)}(k)+P_{ji}^{\left(m\right)}(k)\right)}^{2}}$$ (29)

Dual residual:


$$s^{\left(m\right)}=\rho \sqrt{\sum_{(i,j)\in ℰ}\sum_{k=0}^{H-1}{\left(\phi_{ij}^{\left(m\right)}(k)-\phi_{ij}^{\left(m-1\right)}(k)\right)}^{2}}$$ (30)

Stopping criterion:


$$r^{\left(m\right)}\leq \epsilon_{pri}\;\text{and}\;s^{\left(m\right)}\leq \epsilon_{dual}$$ (31)

For the convex relaxation where binary variables *z*_*i*_(*k*) are relaxed to [0,1], ADMM converges to the global optimum under standard assumptions [8,25]. With binary variables, the problem is nonconvex and standard guarantees do not apply; we treat ADMM as a practical coordination heuristic with warm-starting and iteration limits. Under packet loss (handshake gating and stale updates), we likewise use a practical lossy-update variant and emphasize anytime-feasible execution rather than convergence guarantees.

### Execution policy and anytime feasibility

The physically implemented tie-line value under truncated ADMM and non-reciprocal updates $P_{ij}^{\left(m\right)}\neq -P_{ji}^{\left(m\right)}$ must be specified.

We execute the consensus tie-line schedule using the following policy:

1. At iteration *m* (or termination), the consensus variable $\phi_{ij}^{\left(m\right)}(k)$ is the implemented tie-line setpoint.
2. By construction, $\phi_{ij}^{\left(m\right)}=-\phi_{ji}^{\left(m\right)}$, so the network is always physically consistent.
3. Each microgrid performs a local feasibility repair with $\phi_{ij}^{\left(m\right)}$ fixed as the tie-line schedule.

After ADMM termination at iteration *M*, each microgrid *i* performs a local feasibility-repair solve. Specifically, it solves:


$$\min_{u_{i},\;s}\;\sum_{k=0}^{H-1}(c_{i}(k)\Delta t+\lambda_{LS}\;s_{i}^{LS}(k)\Delta t+\lambda_{SP}\;s_{i}^{SP}(k)\Delta t)$$ (32)

subject to all local constraints with fixed tie-line: $P_{ij}(k)=\phi_{ij}^{\left(M\right)}(k)$ for all $j\in {𝒩}_{i}$, and power balance with slack variables. Here $s_{i}^{LS}(k)$ and $s_{i}^{SP}(k)$ are nonnegative load-shedding and spillage slack variables (defined below). Feasibility here refers to solvability of this soft-constraint repair (slacks may be nonzero); Theorem 1 addresses hard-constraint feasibility when reserve headroom exists and mismatch remains within bounds.

This yields the following anytime properties:

- Network feasibility: Always satisfied (consensus variable is reciprocal by construction)
- Local feasibility: Guaranteed via repair solve with slack variables (soft constraints) [26]
- Optimality: Improves with more iterations under reliable communication for the convex relaxation

### Slack variables with physical interpretation

We introduce separate slack variables with distinct physical meanings:

Load shedding (energy not served) uses slack $s_{i}^{LS}(k)\geq 0$ [kW] with penalty $\lambda_{LS}=1000$ $/kWh.

Spillage / energy dump uses slack $s_{i}^{SP}(k)\geq 0$ [kW] with penalty $\lambda_{SP}=10$ $/kWh. Note that *P*_*spill*,*i*_(*k*) denotes planned renewable curtailment, whereas $s_{i}^{SP}(k)$ is an emergency power-balance slack representing surplus absorption (e.g., dump load) used only when strict balance would otherwise be infeasible.

Modified power balance:


$$\begin{array}{c} \begin{array}{cc} P_{res,i}^{used}(k)+P_{dis,i}(k)-P_{ch,i}(k)+P_{grid,i}^{+}(k)-P_{grid,i}^{-}(k) & \\ \;-\sum_{j\in {𝒩}_{i}}P_{ij}(k)+s_{i}^{LS}(k)-s_{i}^{SP}(k) & \;=P_{load,i}(k) \end{array} \end{array}$$ (33)

Metrics: Energy Not Served (ENS): $\sum_{i,k}s_{i}^{LS}(k)\cdot \Delta t$ [kWh]. Energy Spilled: $\sum_{i,k}s_{i}^{SP}(k)\cdot \Delta t$ [kWh].

### Resilience mechanism

#### Communication failure model.

We consider three types of communication failures:

1. Packet loss: Message from neighbor *j* not received with probability *p*_*loss*_ [27,28]
2. Burst failure: Consecutive losses following Gilbert-Elliott model [29,30]
3. Communication link outage: Communication unavailable on an edge for duration *T*_*fail*_ while the physical tie-line remains intact

Physical tie-line outages (topology changes) are modeled separately by removing the edge (setting *P*_*ij*_ = 0 and updating neighbor sets), and are evaluated in Scenario S6. Scenario S3 uses communication burst outages (tie-lines remain physical but communication is unavailable).

Communication state:


$$\begin{array}{c} \gamma_{ij}^{\left(m\right)}=\left\{ \begin{array}{cc} 1 & \text{message from }j\text{ received by }i\text{ at ADMM iter. }m\\ 0 & \text{otherwise} \end{array}\right. \end{array}$$ (34)

We define a bidirectional handshake indicator $h_{ij}^{\left(m\right)}=\gamma_{ij}^{\left(m\right)}\gamma_{ji}^{\left(m\right)}$.

Unless otherwise noted (e.g., the Path B step-level outage model), packet losses are modeled as independent and identically distributed (i.i.d.) Bernoulli events across directed edges and ADMM iterations.

### ADMM behavior under packet loss

When a message from neighbor *j* is not received at iteration *m*, we apply a stale-update rule:

- Use last received value: $P_{ji}^{\left(m\right)}\leftarrow P_{ji}^{\left(m-1\right)}$ (or last available)
- Track iteration staleness: $\tau_{ij}^{iter}(m)=m-m_{last\_received}$
- Freeze dual update when handshake fails: $\mu_{ij}^{\left(m+1\right)}=\mu_{ij}^{\left(m\right)}$ if $h_{ij}^{\left(m\right)}=0$

We distinguish (i) *iteration staleness* within an ADMM solve (used only for stale-update bookkeeping) and (ii) *contract staleness* across MPC steps, defined as the number of MPC steps since the last successful handshake on a directed edge. The mismatch bound used for reserve sizing is a function of the contract staleness $\tau_{ij}^{step}(t)$.

As contract staleness increases, we inflate and saturate the mismatch bound:


$${\bar{w}}_{ij}(\tau^{step})=\min\left\{{\bar{w}}_{ij}^{max},\;\max\left\{{\bar{w}}_{ij}^{min},\;{\bar{w}}_{ij}^{base}+\alpha_{stale}\;\tau_{ij}^{step}\right\}\right\}.$$ (35)

where $\alpha_{stale}$ reflects uncertainty growth with staleness.

### Bounded mismatch model

Under communication failure, microgrid *i* cannot coordinate with neighbor *j*. The planned tie-line exchange may not match the neighbor’s actual behavior.

Realized tie-line power:


$${\tilde{P}}_{ij}(k)=P_{ij}^{plan}(k)+w_{ij}(k)$$ (36)

where *w*_*ij*_(*k*) is the mismatch disturbance, bounded by $|w_{ij}(k)|\leq {\bar{w}}_{ij}$. We set $P_{ij}^{plan}(k):=\phi_{ij}^{\left(M\right)}(k)$ during execution (the executed contract setpoint).

### Two-sided constraint tightening

Mismatch can cause either a deficit (needs upward reserve) or a surplus (needs downward reserve). Both directions must be protected. We implement tightening following robust/tube MPC principles [31].

Upward reserve (can increase net injection) must satisfy:


$$R_{i}^{up}(k)\geq \sum_{j\in {𝒩}_{i}^{disc}}{\bar{w}}_{ij}$$ (37)

It is provided by ESS discharge headroom, grid import headroom, and additional load curtailment.

Downward reserve (can decrease net injection) must satisfy:


$$R_{i}^{down}(k)\geq \sum_{j\in {𝒩}_{i}^{disc}}{\bar{w}}_{ij}$$ (38)

It is provided by ESS charging headroom, grid export headroom, and renewable spillage.

These reserves impose the following constraints. For upward:


$$\left(P_{dis,i,max}-P_{dis,i}(k))+(P_{grid,i,max}^{+}-P_{grid,i}^{+}(k))+(P_{curt,i,max}(k)-P_{curt,i}(k))\geq R_{i}^{up}(k\right)$$ (39)

For downward:


$$\left(P_{ch,i,max}-P_{ch,i}(k))+(P_{grid,i,max}^{-}-P_{grid,i}^{-}(k))+({\hat{P}}_{res,i}(k)-P_{spill,i}(k))\geq R_{i}^{down}(k\right)$$ (40)

### Operating modes

We describe two coordination regimes based on the set of disconnected neighbors. Mode 1 (Full Coordination, $|{𝒩}_{i}^{disc}|=0$) uses standard ADMM-based DMPC without reserve tightening. Mode 2 (Partial Coordination, $0<|{𝒩}_{i}^{disc}|\leq |{𝒩}_{i}|$) continues ADMM with connected neighbors while applying two-sided reserve tightening for disconnected edges; stale values are held from the last successful exchange. If $\left|{𝒩}_{i}^{disc}|=|{𝒩}_{i}\right|$, coordination reduces to local MPC with a fixed reciprocal tie-line contract (held under packet loss and updated only upon bidirectional handshake), together with tightened reserves.

### Feasibility guarantee

The following sufficient feasibility condition follows from standard robust/tube MPC reasoning for bounded additive disturbances [31]. **Theorem 1 (Feasibility under bounded mismatch).** If the two-sided reserve margins satisfy:


$$R_{i}^{up}(k)\geq \sum_{j\in {𝒩}_{i}^{disc}}{\bar{w}}_{ij},\;R_{i}^{down}(k)\geq \sum_{j\in {𝒩}_{i}^{disc}}{\bar{w}}_{ij}$$ (41)

and sufficient local flexibility exists such that the net adjustable range within ESS/grid/DR/spillage constraints contains $\left[-\sum_{j\in {𝒩}_{i}^{disc}}{\bar{w}}_{ij},\;+\sum_{j\in {𝒩}_{i}^{disc}}{\bar{w}}_{ij}\right]$, then the power balance constraint is satisfiable for any realization of mismatch $|w_{ij}(k)|\leq {\bar{w}}_{ij}$.

*Proof:* Consider the worst cases: (1) Maximum deficit ($w_{ij}=+{\bar{w}}_{ij}$ for all disconnected *j*): The realized tie-line export is larger than planned, creating a local power deficit of $\sum_{j}{\bar{w}}_{ij}$. The upward reserve $R_{i}^{up}(k)$ can cover this. (2) Maximum surplus ($w_{ij}=-{\bar{w}}_{ij}$ for all disconnected *j*): The realized tie-line export is smaller than planned, creating a local power surplus. The downward reserve $R_{i}^{down}(k)$ can absorb this. For intermediate cases, the available reserves interpolate linearly.◻

### Complete algorithm

The complete Resilient ADMM-DMPC procedure is presented below.

**Algorithm 1: Resilient ADMM-DMPC**

**Input:** Current states {*E*_*i*_(*t*)}, Forecasts {*Pˆ_res,i_, P_fixed,i_*, prices}, Comm. state indicators $\left\{\gamma_{ij}^{\left(m\right)}\right\}$

**Output:** Control actions $\left\{u_{i}^{*}(0)\right\}$

**Parameters:** ρ, *M*_*max*_, $\epsilon_{pri}$, $\epsilon_{dual}$, $\lambda_{LS}$, $\lambda_{SP}$

1. **Initialization:** For each microgrid *i*:

• Determine mode based on connected/disconnected neighbor sets

• Initialize: $\phi_{ij}^{\left(0\right)}=P_{ij}^{last}$, $\mu_{ij}^{\left(0\right)}=0$ for $j\in {𝒩}_{i}$

• For $j\in {𝒩}_{i}^{disc}$: hold stale contract estimate ${\hat{P}}_{ij}\leftarrow P_{ij}^{last}$

• Compute reserve requirements: $R_{i}^{up}$, $R_{i}^{down}$ based on mode

2. **ADMM Iterations:** For $m=1,2,\ldots ,M_{max}$:

(2a) **Local Optimization** (parallel): Solve QP with ADMM penalty terms

(2b) **Communication:** Send $\left\{P_{ij}^{\left(m\right)}\right\}$ to $j\in {𝒩}_{i}^{conn}$; Receive $\left\{P_{ji}^{\left(m\right)}\right\}$. If received: $\tau_{ij}^{iter}\leftarrow 0$; else: $P_{ji}^{\left(m\right)}\leftarrow P_{ji}^{\left(m-1\right)}$, $\tau_{ij}^{iter}\leftarrow \tau_{ij}^{iter}+1$. Define handshake $h_{ij}^{\left(m\right)}=\gamma_{ij}^{\left(m\right)}\gamma_{ji}^{\left(m\right)}$

(2c) **Consensus Update:** For each $j\in {𝒩}_{i}$: $\phi_{ij}^{\left(m+1\right)}=\frac{1}{2}(P_{ij}^{\left(m\right)}-P_{ji}^{\left(m\right)})$ if $h_{ij}^{\left(m\right)}=1$; else $\phi_{ij}^{\left(m+1\right)}=\phi_{ij}^{\left(m\right)}$ (hold last)

(2d) **Dual Update:** If $h_{ij}^{\left(m\right)}=1$: $\mu_{ij}^{\left(m+1\right)}=\mu_{ij}^{\left(m\right)}+\rho (P_{ij}^{\left(m\right)}-\phi_{ij}^{\left(m+1\right)})$; else $\mu_{ij}^{\left(m+1\right)}=\mu_{ij}^{\left(m\right)}$ (freeze)

(2e) **Convergence Check:** Compute *r^(m)^*, *s^(m)^*. If $r^{\left(m\right)}\leq \epsilon_{pri}$ and $s^{\left(m\right)}\leq \epsilon_{dual}$: break

3. **Execution:** For each microgrid *i*:

• Set tie-line setpoint: $P_{ij}^{exec}=\phi_{ij}^{\left(M\right)}$ for all *j*

• Solve local repair problem (fix tie-lines, minimize slack)

• Implement first-step actions: $u_{i}^{*}(0)$

• Store $P_{ij}^{exec}(0)$ as $P_{ij}^{last}$ for next interval

4. **Return:** control actions, convergence info, slack usage

**Definition (Handshake).** A *handshake* occurs when both microgrids *i* and *j* successfully exchange their proposed tie-line setpoints *P*_*ij*_ and *P*_*ji*_ within the same ADMM iteration (i.e., $h_{ij}^{\left(m\right)}=1$). The bidirectional exchange is required for contract execution, and upon a successful handshake the consensus variable $\phi_{ij}=-\phi_{ji}$ is updated and used for execution; if either direction fails (asymmetric packet loss), the stale contract from the previous successful handshake is retained (hold-last policy).

**Fig 2:**
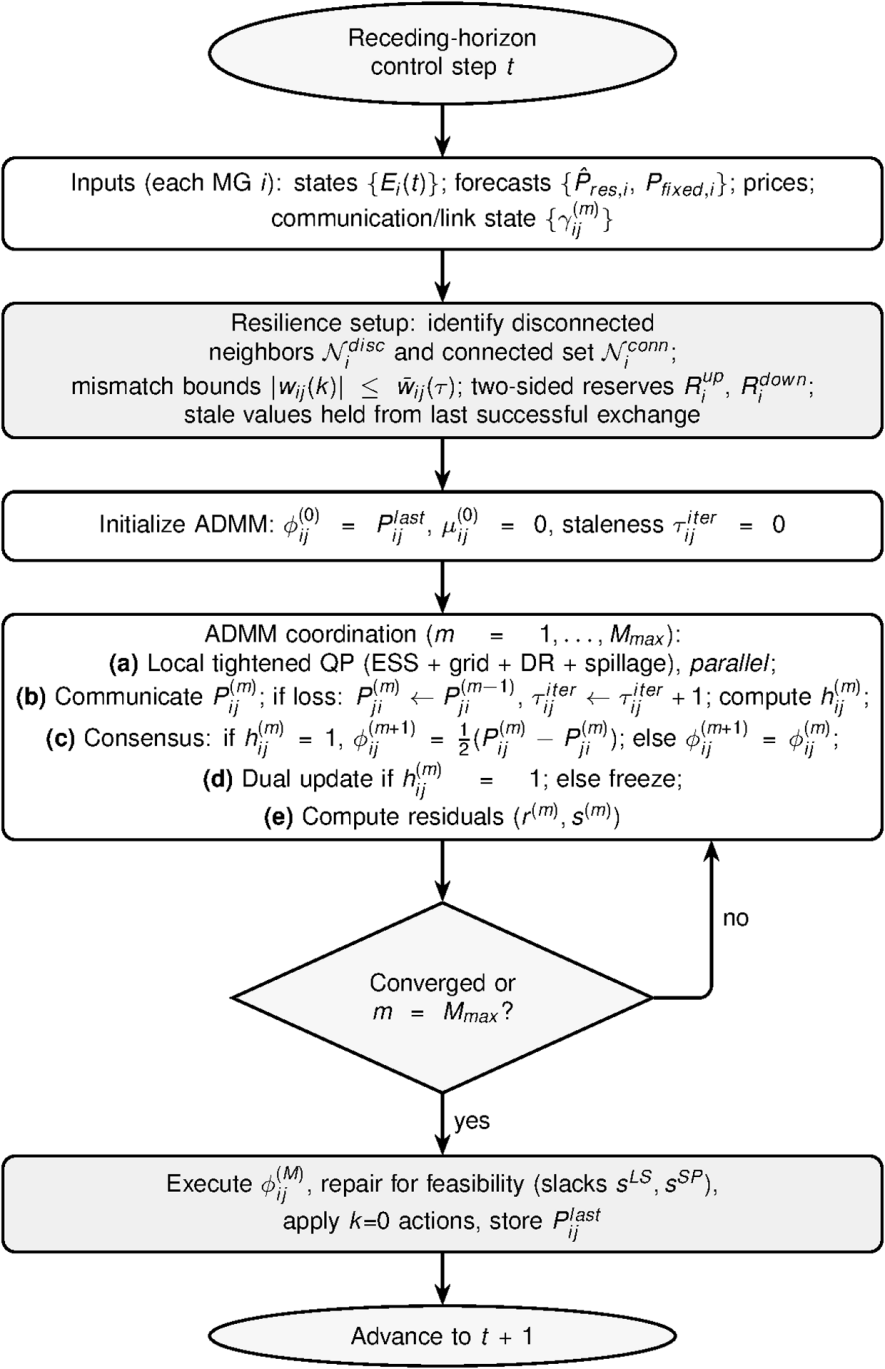
Methodology flowchart of the proposed RDMPC. At each MPC step, microgrids (i) perform mode-dependent resilience setup with bounded tie-line mismatch and two-sided reserve tightening, (ii) coordinate via lossy-communication ADMM, and (iii) execute the reciprocal consensus tie-line schedule with a local feasibility-repair solve, ensuring anytime feasibility even when ADMM is truncated.

Fig 2 provides a visual overview of the procedure and its execution semantics under communication loss.

### Complexity analysis

Per ADMM iteration per microgrid (planning step): QP with $O(H\cdot (7+|{𝒩}_{i}|))$ continuous variables (tie-lines plus local energy variables, including spillage). Solved with CLARABEL in our experiments [32] (OSQP [33] and SCS [34] are compatible alternatives).

Per MPC step per microgrid (execution step): one additional local feasibility-repair QP with the tie-line schedule fixed to the consensus contract, so it has fewer free variables (O(H·7), since tie-line trajectories are parameters rather than decision variables). The repair is solved once per MPC step (not once per ADMM iteration), so its cost is typically small relative to *M*_*max*_ planning solves. As communication impairments grow, the number of MPC steps with nonzero slack may increase, but the repair problem size does not depend on the number of loss events; only the realized fixed tie-line schedule changes.

Communication per iteration: Each microgrid sends $O(H\cdot |{𝒩}_{i}|)$ floats to neighbors. Total network communication: O(H·|ℰ|) floats.

Total computation per MPC step scales as $O(N\cdot M_{max})$ local QP solves for planning plus *O*(*N*) repair solves, parallelizable across microgrids. Total communication per MPC step scales as $O(M_{max}\cdot H\cdot |ℰ|)$ floats. For sparse microgrid graphs where node degree is bounded, |ℰ|=O(N), so communication scales approximately linearly in *N*; for dense topologies, communication can grow as *O*(*N*^2^). ADMM iteration counts can increase with network size and under packet loss due to staleness, motivating an iteration cap; the proposed execution policy maintains feasibility at any iteration, trading optimality for runtime when coordination is slow.

### AI use disclosure

GPT 5.2 via the OpenAI API was used for author-guided drafting and refinement of initial paragraphs in the literature review, Materials and methods, and Results sections; all AI-assisted text was then revised and checked by the author. GPT 5.2 was also used to assist with author-guided edits to the simulation code. All output was author-verified against primary sources.

## Results

### Simulation design

All reported experiments use the convex QP relaxation (binary ESS mode relaxed) and are solved with CLARABEL [32]. OSQP [33] and SCS [34] are compatible alternatives.

We compare against the following baselines in all experiments:

1. B1 (Oracle planning benchmark; loss-unaware DMPC): ADMM-based DMPC planning assuming reliable communication; execution uses the same communication loss realizations and contract/repair policy as B2 and B3 for comparability (not deployable under loss).
2. B2 (Naive DMPC): ADMM-based DMPC with stale consensus/dual updates when packets drop during planning, but no resilience mechanisms (reserves or constraint tightening).
3. B3 (Proposed RDMPC): Full method with two-sided reserves, staleness-aware constraint tightening, and contract execution policy.

All methods share the same contract execution and local feasibility-repair policy; B3 uniquely applies reserve tightening during planning.

Execution semantics by method are summarized in Table 1.

**Table 1:** Execution semantics by method.

Method	Contract execution (ϕ)	Local repair (slacks)	Reserve tightening
B1	Yes	Yes	No
B2	Yes	Yes	No
B3	Yes	Yes	Yes

Reported experimental scenarios are summarized in Table 2.

**Table 2:** Experimental scenarios evaluated in Results.

Scenario	Description	Parameters
S2: Random loss.	Bernoulli packet loss	*p_loss_* = 0.1–0.5
S3: Burst outage.	Critical edges forced down after burn-in	*T*_*fail*_ = 3 (steps 3–5)
S6: Topology change.	Link removed permanently at step 3	*p*_*loss*_ = 0.3
S7: DR ablation.	DR disabled (shiftable/curtailable fractions set to zero)	*p*_*loss*_ = 0.3

Scenario numbering follows the codebase convention; only scenarios evaluated in Results are listed here.

We report the following performance metrics:

- Economic: Executed operating cost [$]
- Feasibility: Energy Not Served (ENS) [kWh], curtailment [kWh], spillage (renewable spillage) [kWh]
- Coordination/communication: contract staleness (max/mean, in MPC steps), contract update rate, directed and handshake loss rates
- Resilience: Relative performance under failures and DR ablation effects

Table 3 lists nominal (Path A) simulation parameters.

**Table 3:** Nominal (Path A) simulation parameters.

Parameter	Symbol	Value	Unit
Number of microgrids	*N*	5	–
Prediction horizon	*H*	24	hours
Control interval	Δt	1	hour
ESS capacity	*E*_*max*_	500	kWh
ESS power limit	*P*_*ess*,*max*_	250	kW
Charging efficiency	$\eta_{c}$	0.95	–
Discharging efficiency	$\eta_{d}$	0.95	–
PV capacity	*P*_*pv*,*rated*_	200	kW
Wind capacity	*P*_*wt*,*rated*_	150	kW
Max grid import	$P_{grid,max}^{+}$	100	kW
Max grid export	$P_{grid,max}^{-}$	100	kW
Max tie-line	*P*_*ij*,*max*_	100	kW
ADMM penalty	ρ	5	p.u.
Max iterations	*M*_*max*_	20	–
ESS wear penalty	$\lambda_{ess}$	1	$/kWh
DR shift penalty	$\lambda_{shift}$	0.1	$/kWh
DR curtail penalty	$\lambda_{curt}$	5	$/kWh
Renewable spillage penalty	$\lambda_{spill}$	0.01	$/kWh
Load shedding penalty	$\lambda_{LS}$	1000	$/kWh
Spillage slack penalty	$\lambda_{SP}$	10	$/kWh
Base mismatch bound	${\overset{―}{\omega }}_{ij}^{base}$	0	kW
Minimum mismatch bound	${\overset{―}{\omega }}_{ij}^{min}$	2	kW
Maximum mismatch bound	${\overset{―}{\omega }}_{ij}^{max}$	50	kW
Staleness growth factor	$\alpha_{stale}$	1	kW/step

*Note (Path B overrides to Table 3):* Path B uses asymmetric donor/receiver limits (donors: $P_{grid,max}^{-}=20$ kW, *P*_*ess*,*max*_ = 250 kW; receivers: $P_{grid,max}^{+}=60$ kW, $P_{grid,max}^{-}=40$ kW, *P*_*ess*,*max*_ = 70 kW; neutral MGs: $P_{grid,max}^{\pm }=80$ kW, *P*_*ess*,*max*_ = 150 kW), a higher tie-line limit (150 kW), and an iteration cap of *M*_*max*_ = 15. The S6 topology-change scenario uses a tighter receiver configuration ($P_{grid,max}^{+}=10$ kW, *P*_*ess*,*max*_ = 50 kW).

## Results overview

We report results in two operating regimes: (i) a coordination-critical loss-phase regime (Path B) and (ii) a grid-connected economic regime (Path A). Path B evaluates a setting in which communication failures can produce infeasible local schedules unless resilience and anytime-feasible execution are enforced; this regime directly tests the proposed bounded-mismatch tightening and execution policy. Path A evaluates a grid-connected regime with negligible ENS, serving as a no-harm check that resilience mechanisms do not introduce systematic cost penalties under packet loss. We present Path B first because it is the primary stress test of resilience, then the S7 ablation (demand response contribution), and finally Path A as the economic no-harm check.

### Path B: Loss-phase execution under contract loss

We evaluate a coordination-critical regime using a load pattern that tightens receiver-side constraints, with a 3-step initialization phase (no packet loss) followed by 5 steps under communication impairment (total 8 steps). Packet loss is generated by the step-level outage model with $p_{loss}(=p_{b})=0.3$ (directional loss). Results report loss-phase only (steps 3–7). We distinguish *directional loss* (a one-way packet drop on an edge) from *handshake loss* (failure of the bidirectional exchange required to establish a reciprocal tie-line contract for execution). Over 20 seeds, the loss-phase mean rates are 15.75% directional and 29.5% handshake. Here *p*_*loss*_ corresponds to the bad-state loss probability *p*_*b*_ in the Gilbert–Elliott step-level model (with *p*_*g*_ = 0.05, *p*_*b*_ = 0.3, $p_{gb}=p_{bg}=0.2$), so the observed rates are step-level directed-edge averages rather than i.i.d. per-iteration packet loss. These parameters are representative engineering settings chosen to yield moderate step-level directed-edge and handshake loss rates (reported above), rather than calibrated from a specific field communication trace. Staleness metrics reported below are contract staleness across MPC steps (number of MPC steps since the last successful handshake on a directed edge), summarized as the maximum across edges per step and then averaged over the loss phase.

B1 (oracle planning) uses the same execution policy under the same communication loss realizations but plans as if all packets succeed; it lacks resilience mechanisms (no two-sided reserves or staleness-based tightening), serving as an optimistic planning baseline. It is included as an upper-bound reference and is not deployable under lossy communication.

Table 4 shows loss-phase results.

**Table 4:** Path B results: Loss-phase execution metrics (per-seed totals over 5 steps, n = 20 seeds).

Method	Exec ENS	Exec Cost	Exec Curtail	Exec Spill	Max stale	Mean max stale
	(kWh)	($)	(kWh)	(kWh)	(MPC steps)	(MPC steps)
B1 (oracle planning)	4.2	5,707	61.3	0.00	–	–
B2 (naive)	84.4	85,961	90.5	0.00	5	2.20
B3 (RDMPC)	86.6	88,101	90.3	0.00	5	2.20

Relative to B2, B3 does not consistently reduce executed ENS or cost in this handshake-gated setting. Paired per-seed deltas are mixed: B3 improves ENS in 5/20 seeds (5 ties, 10 worse) and improves cost in 10/20 seeds (10 worse). Mean deltas are small ($\Delta_{\text{ENS}}\approx +2.14$ kWh, $\Delta_{\text{cost}}\approx +\$2.14\text{k}$), indicating seed-dependent outcomes rather than a uniform improvement. Formal paired tests on the 20 per-seed loss-phase deltas corroborate the absence of consistent dominance: two-sided Wilcoxon signed-rank tests yield *p* = 0.216 (ENS) and *p* = 0.622 (cost), and paired t-tests yield *p* = 0.320 (ENS) and *p* = 0.320 (cost). The unique contribution of B3 in Path B is the hard-constraint feasibility guarantee under bounded mismatch when reserve headroom exists (Theorem 1), while contract execution and local repair are shared across methods. Across loss-phase steps, the max contract staleness exceeded zero in 73% of steps under random loss and 90% under burst outage; the corresponding mean max staleness values are reported in Tables 4–5 (2.20 and 3.60 MPC steps, respectively), with a maximum of 5. In a 5-seed pilot diagnostic (25 loss steps; 125 MG-steps), upward reserve requirements were nonzero in 20% (random loss) and 58% (burst) of MG-steps; binding (headroom ≤ requirement, i.e., positive shortfall slack) occurred in 0.8% and 4.8% of MG-steps, respectively, while downward reserve binding was not observed. Executed spillage is negligible ($<10^{-6}$ kWh; rounded to 0.00 in Tables 4–6) and denotes renewable spillage $\sum_{i,k}P_{spill,i}(k)\Delta t$. Because $\lambda_{LS}=1000$ $/kWh, Path B executed cost is dominated by ENS (e.g., 86.6 kWh corresponds to ∼ $86.6k of the $88.1k mean cost for B3).

**Table 5:** Burst outage results: Loss-phase metrics (*T*_*fail*_ = 3 on critical edges, n = 20 seeds).

Method	Exec ENS	Exec Cost	Exec Curtail	Exec Spill	Max stale	Mean max stale
	(kWh)	($)	(kWh)	(kWh)	(MPC steps)	(MPC steps)
B1 (oracle planning)	4.4	5,910	64.1	0.00	–	
B2 (naive)	50.6	52,156	88.2	0.00	5	3.60
B3 (RDMPC)	47.6	49,117	87.8	0.00	5	3.60

**Table 6:** Scenario S7 (DR ablation): loss-phase execution metrics under step-level random loss (n = 20 seeds). Loss phase is steps 3–7 (5 steps) after a 3-step burn-in; values are loss-phase totals per seed, averaged across seeds. B3-noDR disables DR flexibility (shiftable and curtailable fractions set to zero). Cost in $.

Method	Exec ENS	Exec Cost	Exec Curtail	Exec Spill
	(kWh)	($)	(kWh)	(kWh)
B1 (oracle planning)	4.2	5,707	61.3	0.00
B2 (naive)	84.4	85,961	90.5	0.00
B3 (RDMPC)	86.6	88,101	90.3	0.00
B3-noDR	512.7	514,112	0.0	0.00

### Burst outage on critical edges

We repeat the experiment with a correlated failure pattern: after burn-in (steps 0–2), we enforce a contiguous burst outage on critical donor-receiver tie-lines for steps 3–5 (*T*_*fail*_ = 3), disabling both ADMM coordination and handshake execution on those edges. Steps 6–7 revert to nominal loss model with *p*_*loss*_ = 0.3.

Table 5 shows burst outage results.

**Fig 3:**
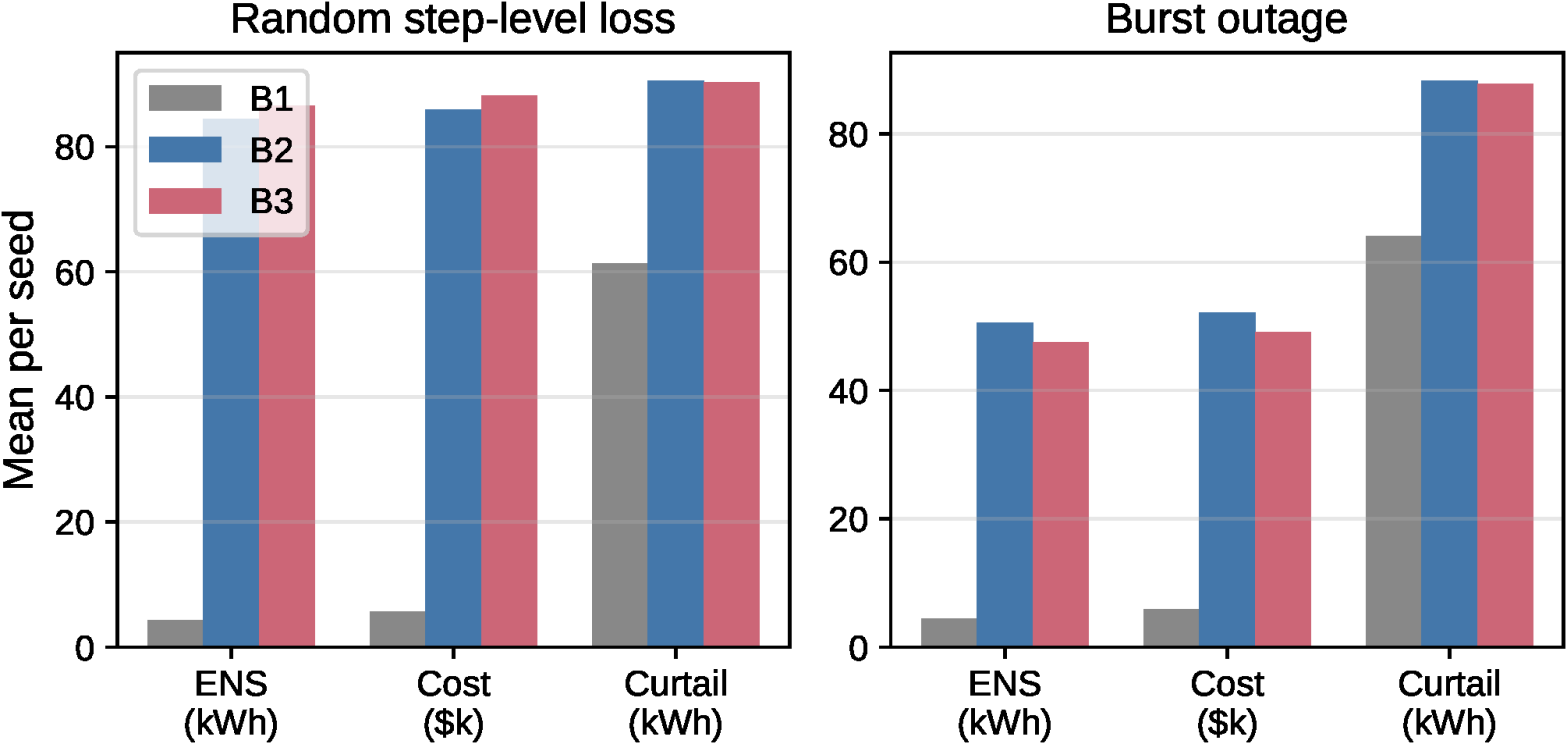
Path B loss-phase mean metrics (n = 20 seeds, loss-phase only). Grouped bar chart comparing B1 (oracle planning), B2 (naive DMPC), and B3 (RDMPC) for executed ENS, cost (shown in $k), and curtailment. Left: random step-level losses (Gilbert–Elliott; directional mean 15.75%, handshake ~29.5%). Right: Burst outage (*T*_*fail*_ = 3 on critical edges). B3 outcomes are mixed relative to B2; improvements are seed-dependent rather than uniform.

Burst outages produce heterogeneous outcomes across seeds; mean differences are modest and not uniform. Formal paired tests on the 20 per-seed deltas again indicate no consistent dominance: two-sided Wilcoxon signed-rank tests yield *p* = 0.898 (ENS) and *p* = 0.674 (cost), and paired t-tests yield *p* = 0.339 (ENS) and *p* = 0.339 (cost). Fig 3 visualizes these loss-phase mean metrics. Given this heterogeneity, we interpret the proposed framework primarily as an execution-safe coordination approach (contract execution + repair), while reserve tightening (B3) is aimed at providing a hard-constraint feasibility guarantee under bounded mismatch when reserve headroom exists (Theorem 1) rather than guaranteeing ENS/cost improvements.

**Fig 4:**
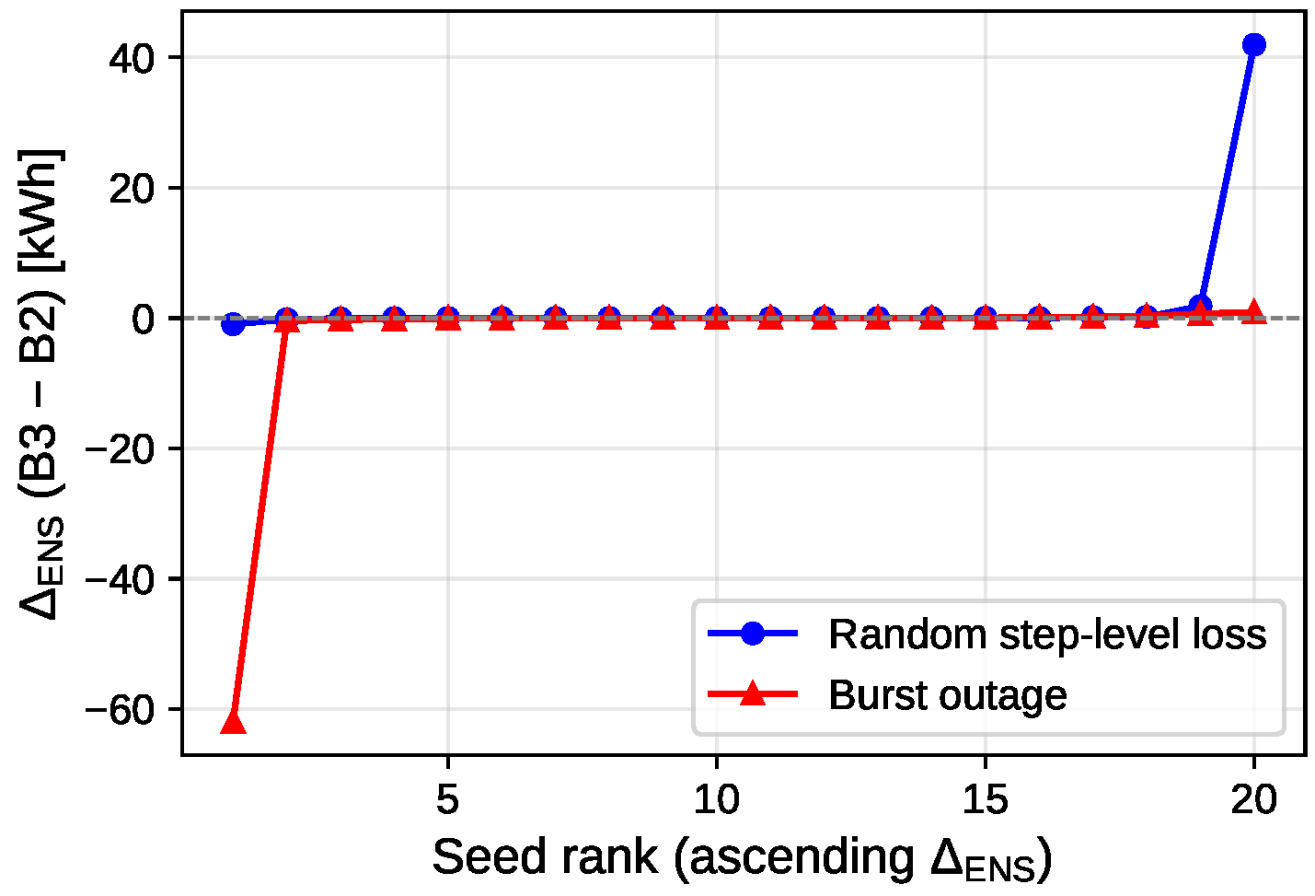
Sorted per-seed executed ENS improvement (loss-phase only, n = 20 seeds). Delta values $\Delta_{\text{ENS}}=\text{B3}-\text{B2 }$ sorted ascending; each curve sorted independently (x-axis is sorted rank position 1,…,n, not the random seed index). Negative values indicate B3 outperforms B2. Under random step-level loss (blue), deltas are mixed (5/20 better, 5 ties, 10 worse), indicating no uniform improvement. Under burst outage (red), outcomes remain heterogeneous (7/20 better, 5 ties, 8 worse), with no consistent dominance.

Fig 4 shows the per-seed deltas; outcomes are heterogeneous across seeds, so gains are not uniform across realizations. This supports a cautious interpretation: the framework ensures feasible reciprocal execution under loss via contract execution and repair, while reserve tightening does not guarantee lower executed ENS/cost for every seed.

### Scenario S7: Demand response ablation under step-level random loss (Path B)

To isolate the contribution of demand response (DR) as a resilience resource (C3), we repeat the Path B random-loss experiment using the proposed controller but *with DR disabled* (denoted B3-noDR). In B3-noDR, shiftable and curtailable load flexibility is disabled (both fractions set to zero), removing all DR flexibility. All other elements are unchanged (same network, forecasts, reserve tightening, ADMM settings, execution policy, and local feasibility repair with slack penalties). The experiment uses the same 20 seeds, with a burn-in of 3 loss-free steps followed by a 5-step loss phase (steps 3–7) under the step-level outage model with *p*_*loss*_ = 0.3. Metrics below report loss-phase totals per seed, averaged across seeds.

Loss-phase metrics for the DR ablation are summarized in Table 6.

**Fig 5:**
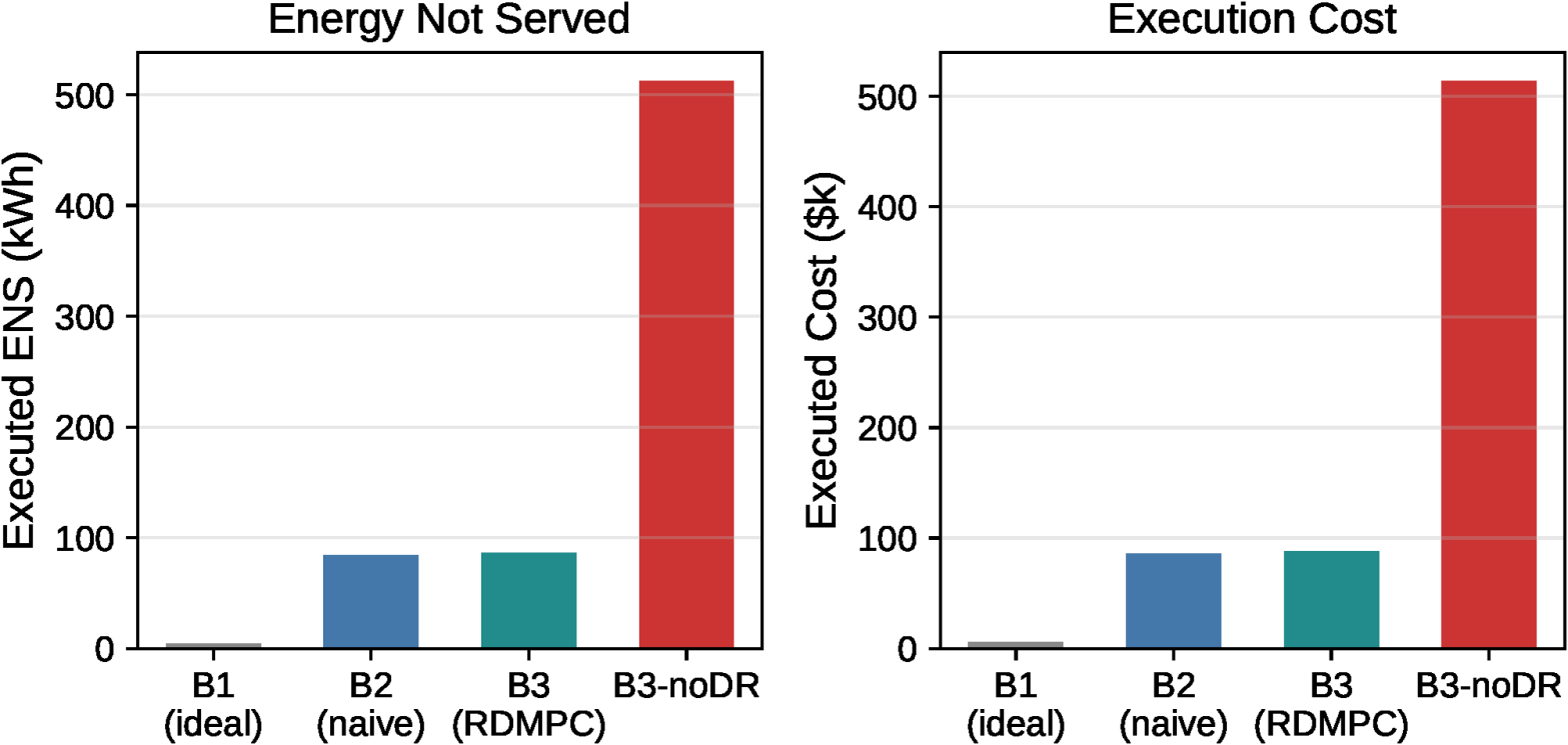
DR ablation: loss-phase execution metrics (n = 20 seeds, per-seed means). Two-panel bar chart comparing B1 (oracle planning), B2 (naive DMPC), B3 (RDMPC with DR), and B3-noDR (RDMPC without DR). Left: Executed ENS (kWh). Right: Executed cost ($k). Disabling DR increases ENS by 492% (513 vs 86.6 kWh) and cost by 484% ($514k vs $88k).

Disabling DR causes a substantial degradation in loss-phase execution performance. Relative to B3, B3-noDR increases executed energy-not-served (ENS) from 86.6 kWh to 512.7 kWh (a **492%** increase; ~5.92×) and increases executed cost from $88,101 to $514,112 (a **484%** increase; ~5.84×). The paired per-seed deltas (B3 – B3-noDR) are consistently negative across all seeds (20/20): the mean ENS reduction is $\Delta_{\text{ENS}}=-426.15$ kWh with a 95% CI [−508.87, −350.79], and the mean cost reduction is $\Delta_{\text{cost}}=-$ $426,011 with a 95% CI [−$508,595, −$350,771]. Two-sided paired tests confirm the DR benefit is statistically significant (Wilcoxon signed-rank: $p=1.9\times 10^{-6}$ for both ENS and cost; paired t-test: $p\approx 3.2\times 10^{-9}$). As expected, B3-noDR exhibits near-zero executed curtailment by construction, confirming that DR flexibility is removed. The large cost increase is primarily driven by the increased ENS (and the associated load-shedding penalty), indicating that DR provides critical fast flexibility to maintain service under the same communication impairments and reserve-tightened operation (Fig 5).

### Scenario S6: Topology change under step-level random loss (Path B)

To evaluate RDMPC resilience under network reconfiguration, we simulate a topology change where the middle tie-line (between MG3 and MG4 in the 5-MG line topology) is permanently removed at step 3. This models an intentional islanding event or line failure during operation. The topology change triggers immediate neighbor list updates for affected microgrids and clears stale ADMM variables. The experiment uses the same 20 seeds, with a 3-step burn-in (full topology) followed by a 5-step loss phase (steps 3–7) under reduced topology and step-level outage (*p*_*loss*_ = 0.3). Metrics report loss-phase totals per seed, averaged.

Loss-phase metrics for the topology-change scenario are summarized in Table 7.

**Table 7:** Scenario S6 (Topology change): loss-phase execution metrics (n = 20 seeds). Middle tie-line between MG3 and MG4 removed at step 3; loss-phase totals per seed, averaged.

Method	Exec ENS	Exec Cost	Exec Curtail
	(kWh)	($)	(kWh)
B1 (oracle planning)	13.7	15,289	99.0
B2 (naive)	87.9	89,519	106.5
B3 (RDMPC)	87.9	89,519	106.6

Paired deltas (B3 - B2) for the topology change scenario are effectively zero and not statistically significant (two-sided Wilcoxon: *p* = 0.363 for ENS and *p* = 0.936 for cost; paired t-test: *p* = 0.871 for ENS and *p* = 0.925 for cost): $\Delta_{\text{ENS}}=-0.00$ kWh with 95% CI [−0.01, + 0.01]; $\Delta_{\text{cost}}=-\$0.30$ with 95% CI [−$7.07,  + $5.89]; $\Delta_{\text{curt}}=+0.06$ kWh with 95% CI [−0.01, + 0.17]. In contrast, B3 vs B1 shows significantly higher ENS and cost (CIs exclude zero): $\Delta_{\text{ENS}}=+74.22$ kWh with CI [+43.68, + 109.28], $\Delta_{\text{cost}}=$ +$74,230 with CI [+$43,678, + $109,258], while curtailment is modestly higher but not significant ($\Delta_{\text{curt}}=+7.57$ kWh, CI [−5.19, + 21.74]).

**Fig 6:**
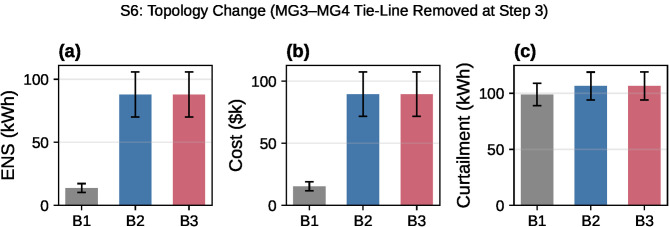
S6: Topology-change impact on B1/B2/B3 performance. Loss-phase totals (steps 3–7) under topology change where the MG3–MG4 tie-line is removed at step 3. Error bars show standard error (SE = std/$\sqrt{n}$) across 20 seeds. B3 (RDMPC) is comparable to B2 in ENS and cost, while B1 remains lowest on average.

These results indicate that, under topology change, handshake-gated RDMPC maintains feasible reciprocal execution but does not yield a consistent performance advantage over B2 in loss-phase ENS/cost (Fig 6).

### Path A: Economic cost parity under packet loss

We evaluate a grid-connected economic regime (ENS ≈ 0) with packet-loss rates *p*_*loss*_ in {0.1, 0.2, 0.3, 0.4, 0.5} and 50 seeds each. Cost excess is defined as ${\text{cost}}_{lossy}-{\text{cost}}_{B1,p=0}$. We report paired differences per seed, Δ=(B3 excess−B2 excess), so negative Δ indicates B3 is better. All costs reported are executed costs, accumulated from the implemented (*k* = 0) actions each step.

For context, a centralized oracle benchmark at *p*_*loss*_ = 0 provides a lower-bound reference; the oracle planning benchmark (B1) remains a strong but not globally optimal reference.

Table 8 shows the paired summary results.

**Table 8:** Path A results: Cost parity under packet loss (n = 50 seeds per *p*_*loss*_).

*p*_*loss*_	mean excess (B2)	mean excess (B3)	mean Δ	95% CI	median Δ
0.10	85.54	85.54	0.00	[-0.00, +0.00]	0.00
0.20	160.96	160.96	0.00	[-0.00, +0.00]	0.00
0.30	273.64	273.64	0.00	[-0.00, +0.00]	0.00
0.40	396.09	396.09	0.00	[-0.00, +0.00]	0.00
0.50	523.04	523.04	0.00	[-0.00, +0.00]	0.00

**Fig 7:**
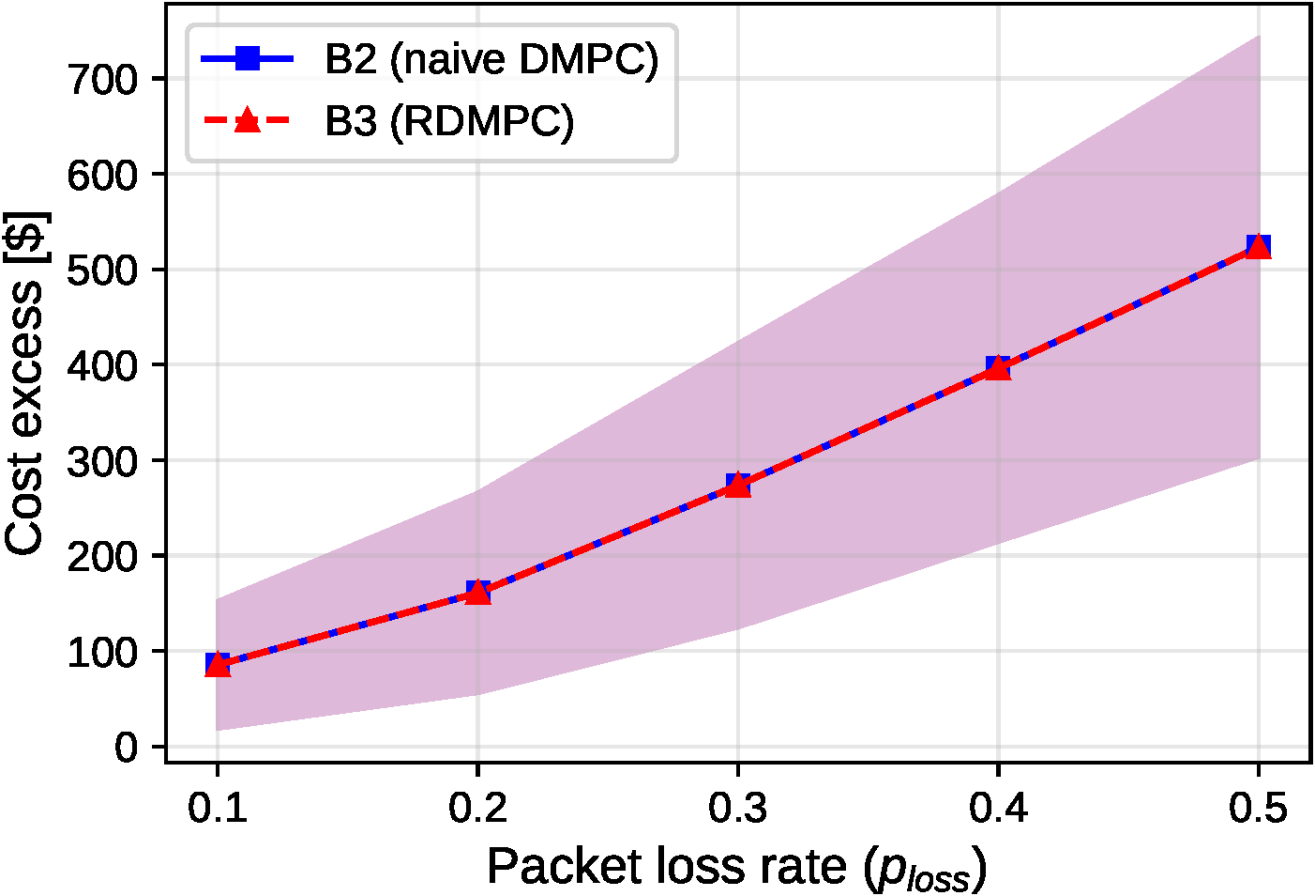
Path A: Cost excess vs. packet loss rate (n = 50 seeds per point). Mean cost excess for B2 (naive DMPC) and B3 (RDMPC) across $p_{loss}\in \{0.1,0.2,0.3,0.4,0.5\}$. Shaded regions show ±1 standard deviation across seeds (not confidence intervals). Lines overlap within variability bands at all loss rates, confirming cost parity.

Mean cost excess versus packet loss is summarized in Fig 7.

**Fig 8:**
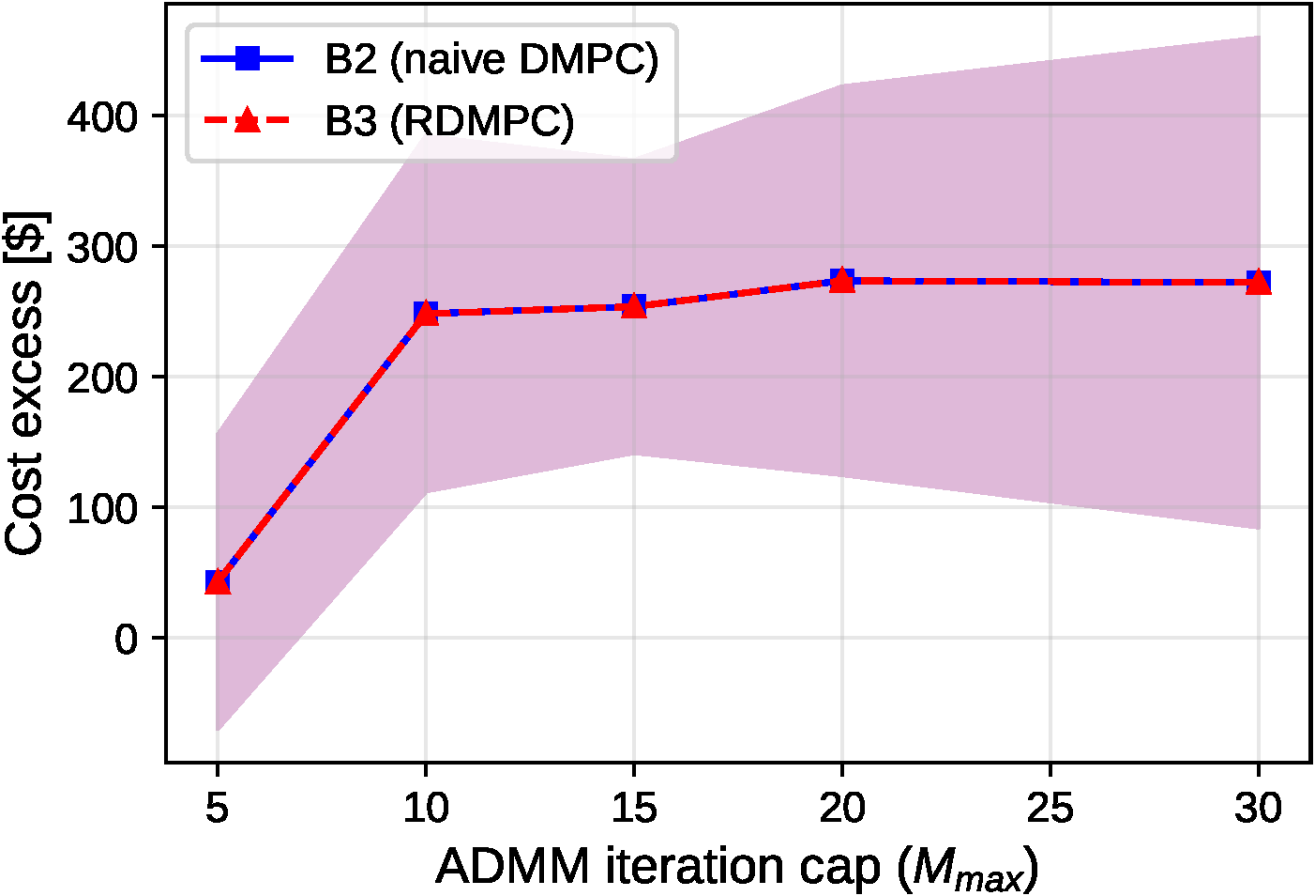
Path A: Anytime behavior vs. iteration budget. Mean cost excess versus ADMM iteration budget *M*_*max*_ (n = 50 seeds, *p*_*loss*_ = 0.3). Under packet loss, larger *M*_*max*_ increases cost excess and then saturates, reflecting a tradeoff between coordination effort and cumulative loss exposure; feasibility remains anytime via the execution/repair policy.

The iteration-cap tradeoff is shown in Fig 8.

Fig 7 shows these results; paired bootstrap CIs for the mean Δ are effectively zero at all *p*_*loss*_ (within $\pm 10^{-4}$ $, rounded to 0.00 in Table 8). Fig 8 shows that, in this lossy-update setting, larger *M*_*max*_ does not yield monotonic improvement in cost excess and can increase it by increasing communication exposure. The anytime property here refers to feasibility (network-consistent reciprocal execution with local repair at any iteration), while optimality improvements with additional iterations hold under reliable communication for the convex relaxation. In this grid-connected Path A regime, reserve tightening was inactive in our simulations ($|{𝒩}_{i}^{disc}|=0$ during planning), so B3 reduces to B2 and the resulting executed costs coincide.

## Discussion

### Theoretical contributions

The proposed RDMPC framework provides a formal feasibility guarantee for hard constraints under bounded communication failures through two-sided reserves, assuming sufficient reserve headroom; the execution repair guarantees feasibility with slack variables when mismatch exceeds bounds. The characterization of the performance-resilience tradeoff enables principled sizing of reserve margins. The execution policy ensures network-consistent tie-line schedules at any ADMM iteration, providing anytime feasibility regardless of convergence status.

### Practical contributions

The algorithm is implementable with standard optimization solvers (CLARABEL used here; OSQP and SCS are alternatives). The quantified value of demand response under coordination failures shows that DR compensates for lost coordination capability. Guidelines for reserve margin sizing based on staleness and mismatch bounds enable practical deployment.

In practice, the two-sided tie-line mismatch bounds ($-{\bar{w}}_{ij}\leq w_{ij}(k)\leq {\bar{w}}_{ij}$) should reflect a maximum credible reciprocity error on each tie-line during a communication impairment. A system operator can choose ${\bar{w}}_{ij}^{max}$ based on tie-line ratings and historical/engineering limits on how far the realized intertie flow could deviate from the last agreed contract during a loss event, and tune the growth factor $\alpha_{stale}$ to match how quickly uncertainty grows with contract staleness. The proposed implementation uses a staleness-dependent bound with a deadband (${\bar{w}}_{ij}^{min}$) and cap (${\bar{w}}_{ij}^{max}$) (Table 3), so tightening activates only when a handshake fails (contract staleness $\tau_{ij}>0$). Very conservative caps can reduce economic performance under prolonged loss by shrinking the feasible region via larger reserve margins; however, they do not penalize normal operation because reserve tightening sums only over disconnected neighbors (${𝒩}_{i}^{disc}=\emptyset$ when all handshakes succeed), so no reserve requirement is imposed; in the grid-connected economic regime (Path A) reserve tightening did not activate and B3 remained cost-parity with B2.

## Experimental findings

Path B highlights resilience under coordination-critical conditions. Under random step-level loss, RDMPC maintains feasible reciprocal execution but exhibits seed-dependent performance relative to B2 (better in some seeds, worse in others). Under burst outages, outcomes remain heterogeneous; RDMPC can mitigate severe execution-level degradation in a subset of realizations without uniformly dominating B2.

Whether B3 (RDMPC) performs better than B2 in a given realization is primarily driven by whether reserve tightening becomes active and binds. Tightening is more likely under longer burst outages (higher contract staleness) and when local headroom is limited (e.g., low ESS state of charge and tight grid limits at the time of failure, or high net-load/renewable ramps), which increases the chance that bounded mismatch would otherwise violate hard constraints. In contrast, when local flexibility is ample or loss episodes are short/mild, tightening rarely binds and B3 is effectively neutral relative to B2.

The DR ablation study isolates the contribution of demand response: disabling DR increases ENS by 492% (~5.92×) and executed cost by 484% (~5.84×) under the same communication failure conditions. This confirms that DR acts as a local flexibility resource that compensates for tie-line mismatch when coordination degrades.

Path A results support a no-harm claim: RDMPC is cost-parity with naive DMPC under packet loss in grid-connected economic regimes, with differences indistinguishable at n = 50.

## Limitations and future work

The current evaluation uses a specific test system (5 microgrids with sparse topology); scalability to larger networks requires further study, particularly regarding ADMM iteration counts and communication overhead as |ℰ| grows. Communication-loss parameters for the step-level Gilbert–Elliott model were selected as representative engineering settings (to yield moderate loss rates) rather than calibrated from field communication traces.

All reported experiments use the convex QP relaxation (binary ESS mode relaxed), so convergence issues associated with mixed-integer switching were not observed. In a spot-check of the CLARABEL QP solutions under Path B full settings (20 seeds; burn-in=3, steps = 8, max iterations = 15, *p*_*loss*_ = 0.3), simultaneous ESS charge/discharge above 10^−4^ kW did not occur (max $\min(P_{ch},P_{dis})\approx 9.8\times 10^{-5}$ kW), suggesting the relaxation does not materially activate simultaneous charge/discharge in practice for the studied regimes. However, under more stressed operating conditions the relaxation could in principle admit fractional modes; evaluating full MIQP formulations (and their convergence/anytime behavior under loss) remains future work. Future work should also explore adaptive ADMM penalty selection and dynamic reserve sizing based on observed communication quality.

## Conclusion

This paper has presented a resilient distributed model predictive control framework designed for networked microgrids with demand response integration. Three key contributions emerge from this work: (1) an ADMM-based coordination scheme for which standard convergence results apply under reliable communication in the convex relaxation, together with an execution policy providing anytime feasibility; (2) a resilience mechanism that models tie-line mismatch as bounded disturbances and applies two-sided constraint tightening, guaranteeing feasibility when mismatch remains within assumed bounds and sufficient reserve headroom exists; and (3) the inclusion of both shiftable and curtailable demand response as additional flexibility resources.

The simulation results show that RDMPC maintains feasible reciprocal execution under random step-level loss and burst outages in coordination-critical conditions (Path B), while performance relative to B2 is seed-dependent rather than uniformly better. The demand response ablation study confirms the substantial resilience value of DR: removing DR flexibility causes large increases in ENS and executed cost under identical failure conditions. Under grid-connected economic regimes (Path A), RDMPC maintains cost parity with naive DMPC (B2), with differences indistinguishable at n = 50.

The proposed framework provides a principled approach to resilient microgrid coordination that maintains feasibility under communication failures, conditioned on bounded mismatch and sufficient reserve headroom (Theorem 1), without sacrificing economic performance under normal operation.

## Supporting information

S1 Table Notation summary. Complete list of symbols used in the paper with descriptions and units.
(S1_Table.PDF)

S1 Data Supporting data package. ZIP archive (S1_Data.zip) containing the processed result summaries (JSON) used to generate the manuscript figures and tables (Path A/B, S6, S7), including paired deltas, confidence intervals, and p-values.
(S1_Data.ZIP)

S2 Code Simulation code package. ZIP archive (S2_Code.zip) containing the simulation code and experiment scripts used to generate the raw result files and processed summaries reported in the manuscript.
(S2_Code.ZIP)
