# Correction: Resilient distributed model predictive control for cooperative microgrids under communication loss with demand response integration

**DOI:** 10.1371/journal.pone.0352945

**Published:** 2026-06-30

**Authors:** The PLOS One Staff

## Notice of Republication

This article was republished on June 12, 2026, to correct an error in the Supporting Information files. An incorrect version of S2 Code was published in error. Please download this article again to view the correct version.
