# Supplementary material for: Resilient distributed model predictive control for cooperative microgrids under communication loss with demand response integration
Source: PLoS One. 2026 Apr 8;21(4):e0345857. doi: 10.1371/journal.pone.0345857 (PMC13061206; doi:10.1371/journal.pone.0345857)
Supplement: S1 Table — Complete list of symbols used in the paper with descriptions and units. (S1_Table.PDF) [file pone.0345857.s001.pdf]

S1 Table. Notation summary

Notation summary. Complete list of symbols used in the paper with descriptions and units.

Symbol	Description	Unit
N	Number of microgrids	–
H	Prediction horizon	steps
Δt	Control interval	h
$E_i(k)$	ESS state of charge	kWh
$P_{ch,i}(k)$	ESS charging power	kW
$P_{dis,i}(k)$	ESS discharging power	kW
$z_i(k)$	Binary charge/discharge selector	–
$P^+_{grid,i}(k)$	Grid import power	kW
$P^-_{grid,i}(k)$	Grid export power	kW
$P_{ij}(k)$	Tie-line flow from i to j	kW
$P_{shift,i}(k)$	Shiftable load power	kW
$P_{curt,i}(k)$	Curtailed load power	kW
$P_{spill,i}(k)$	Renewable spillage	kW
$s_i^{LS}(k)$	Load shedding slack	kW
$s_i^{SP}(k)$	Spillage slack	kW
$\phi_{ij}(k)$	ADMM consensus variable	kW
$\mu_{ij}(k)$	ADMM dual variable	\$/kW
ρ	ADMM penalty parameter	p.u.
$\gamma_{ij}^{(m)}$	Communication state (0/1)	–
$w_{ij}(k)$	Tie-line mismatch disturbance	kW
$\bar{w}_{ij}$	Mismatch bound	kW
$\bar{w}_{ij}^{base}$	Base mismatch bound	kW
$\bar{w}_{ij}^{min}$	Minimum mismatch bound	kW
$\bar{w}_{ij}^{max}$	Maximum mismatch bound	kW
α_{stale}	Staleness growth factor	kW/step
$R_i^{up}(k)$	Upward reserve requirement	kW
$R_i^{down}(k)$	Downward reserve requirement	kW
λ_{ess}	ESS wear penalty	\$/kWh
λ_{shift}	Shift deviation penalty	\$/kWh
λ_{curt}	Curtailment penalty	\$/kWh
λ_{spill}	Renewable spillage penalty	\$/kWh
λ_{LS}	Load shedding penalty	\$/kWh
λ_{SP}	Spillage slack penalty	\$/kWh
$c_i(k)$	Instantaneous cost rate	\$/h
$C_i(k)$	Interval cost	\$
